# High Potential Decolourisation of Textile Dyes from Wastewater by Manganese Peroxidase Production of Newly Immobilised *Trametes hirsuta* PW17-41 and FTIR Analysis

**DOI:** 10.3390/microorganisms10050992

**Published:** 2022-05-09

**Authors:** Bancha Thampraphaphon, Cherdchai Phosri, Nipon Pisutpaisal, Pisit Thamvithayakorn, Kruawan Chotelersak, Sarper Sarp, Nuttika Suwannasai

**Affiliations:** 1 Department of Microbiology, Faculty of Science, Srinakharinwirot University, Bangkok 10110, Thailand; bancha.tamprapaporn@g.swu.ac.th (B.T.); pisit.tham@g.swu.ac.th (P.T.); 2 Department of Biology, Faculty of Science, Nakhon Phanom University, Nakhon Phanom 48000, Thailand; cherd.phosri@npu.ac.th; 3 Department of Agro-Industrial, Food and Environmental Technology, Faculty of Applied Science, King Mongkut’s University of Technology North Bangkok, Bangkok 10800, Thailand; nipon.p@sci.kmutnb.ac.th; 4 Department of Microbiology, Faculty of Medicine, Srinakharinwirot University, Bangkok 10110, Thailand; kruawanc@g.swu.ac.th; 5 Centre for Water Advanced Technologies and Environmental Research (CWATER), College of Engineering, Swansea University, Fabian Way, Swansea SA1 8EN, UK; sarper.sarp@swansea.ac.uk

**Keywords:** textile dyes, decolourisation, white-rot fungi, manganese peroxidase, immobilisation, ADMI, FTIR

## Abstract

Coloured wastewater from the textile industry is a very serious global problem. Among 16 different white-rot fungal isolates, *Trametes hirsuta* PW17-41 revealed high potential for decolourisation of mixed textile dyes (Navy EC-R, Ruby S3B and Super Black G) from real industrial wastewater samples. The efficiency of dye decolourisation was evaluated using the American Dye Manufacturers’ Institute (ADMI) standard methodology. The suitable support for fungal mycelium immobilisation was nylon sponges. The optimal dye decolourisation (95.39%) was achieved by using palm sugar and ammonium nitrate as carbon and nitrogen sources, respectively. The initial pH was 5 and the agitation speed was 100 rpm at 30 °C. The ADMI values of textile dyes decreased from 2475 to 114 within two days, reducing the treatment time from seven days before optimisation. The major mechanism of dye decolourisation was biodegradation, which was confirmed by UV–visible and FTIR spectra. Manganese peroxidase (MnP) (4942 U L^−1^) was found to be the main enzyme during the decolourisation process at an initial dye concentration of 21,200 ADMI. The results indicated the strong potential of immobilised fungal cells to remove high concentrations of textile dyes from industrial wastewater and their potential ability to produce high MnP and laccase activities that can be used in further application.

## 1. Introduction

Wastewater from the textile industry is one of the main polluters in industrial wastewaters due to the dyes it contains. There are more than 10,000 different synthetic dyes on the market, which are extensively used in textile dyeing and printing processes. Most synthetic dyes contain heterocyclic, anthraquinone, azo, phthalocyanine and triphenylmethane compounds, which are sustained in wastewater and the environment [1]. The dyes released into wastewater are toxic to the aquatic environment because the dyes can interfere with photosynthesis and decrease the solubility of oxygen gas in the water. Therefore, both chemical and biological oxygen demands are increased, which results in an ecosystem imbalance [2,3]. The effluents from textile industries contain many toxic compounds, including heavy metals, which are difficult to remove from wastewater. The control of dye pollution has been extensively studied via both physical and chemical treatments using several methods, including adsorption, oxidation and Fenton reactions [4]. Some of these methods can be expensive and can produce a large number of byproducts that require further processing, which can create disposal problems [5]. The adsorption processes are not a complete solution due to the pollutants being transferred from one media to another and not being eliminated. Therefore, biological treatments such as using microorganisms to degrade synthetic dyes are being investigated as viable and cost-effective alternatives.

White-rot fungi are considered to be high-potential microorganisms, with distinguishing capabilities and performances in the biodegradation treatment of synthetic dyes. They can produce non-specific extracellular ligninolytic enzymes such as laccase, manganese peroxidase (MnP) and lignin peroxidase (LiP) [6]. They can degrade a wide variety of recalcitrant compounds and complex structures of several synthetic dyes. White-rot fungi form a diverse eco-physiological group containing mainly basidiomycetes and some ascomycetes. Thamvithayakorn et al. [7] studied the ligninolytic enzyme production from 63 strains of white-rot fungi in northeast Thailand and found that most of them belonged to basidiomycetes, with the exception of one isolate, *Pestalotiopsis theae* PP17-19, which was an ascomycete. Most strains produced laccase and MnP but only a few isolates produced LiP, which were *Aleurodiscus* sp., *Coriolopsis aspera*, *C. retropicta*, *Dentipellis parmastoi*, *Ganoderma lingzhi*, *Ganoderma* sp., *Microporus* sp., *Nigrosporus vinosus*, *Pseudolagarobasidium* sp., *Trametes elegans*, *T. hirsuta*, *T. sanguinea* and *Tyromyces xuchilensis*. Although ligninolytic enzymes have potential for many applications, the main problem with using only enzymes is that the enzymes are inactive or unstable during the treatment process. Therefore, the immobilisation method using whole microbial cells has been extensively studied for wastewater treatment. This method can protect cells from the high concentrations of chemical compounds and high pH levels that might be toxic to free cells, and can preserve the biomass for long retention times. The immobilised cells have high potential to remove the toxic compounds faster than free cells, and can be reused in a continuous bioreactor [8,9]. Furthermore, whole cell cultures can continually produce enzymes during treatment processes. Recently, the immobilisation of *Trametes versicolor* in spherical cartridges with wheat bran as a substrate was used to remove azo dyes under a bioreactor system and exhibited a high efficiency of dye decolourisation [10]. The immobilisation of *T. hirsuta* in alginate beads resulted in high laccase production in an airlift bioreactor, and showed a short incubation time for synthetic dye decolourisation of 24 h [11]. In addition, one of the key steps in improving an efficient dye decolourisation system using white-rot fungi is to use nutrients such as carbon and nitrogen sources, which aid the fungal biomass and ligninolytic enzyme production. The production of these enzymes is commonly induced via agitation in submerged liquid culture and via the adjustment of the initial pH of the system [6,12]. Amaral et al. [13] studied the decolourisation of mixed textile dyes (Procion Orange MX-2R, Reactive Orange 4 and Remazol Red 3B) by *T. versicolor* in the presence and absence of glucose, and found that glucose could induce fungal metabolism, which affected the dye decolourisation efficiency. However, most studies have evaluated dye decolourisation using spectrophotometry at the optimal density level of each dye, while real textile wastewater contains several chemical substances, including synthetic dyes and other pigments. The measurement of the American Dye Manufacturers’ Institute (ADMI) values, which cover all wavelengths of visible colours, has been used as an industrial effluent standard in Thailand since 2017 [14]. The Ministry of Industry have stated that the ADMI value of wastewater from the textile industry has to be less than 300 ADMI before being discharged into the environment. Therefore, the aims of the present study were to select a high-potential white-rot fungal strain for dye decolourisation of the real textile wastewater and to study suitable supports for immobilised fungal mycelium. The effects of carbon and nitrogen sources and the initial pH and agitation speed for textile dye decolourisation were investigated. The ligninolytic enzyme production by the white-rot fungus was then observed. Lastly, the mechanism involved in the dye decolourisation was confirmed via UV–visible and FTIR spectra.

## 2. Materials and Methods

### 2.1. Textile Dyes and Chemicals

The textile wastewater was obtained from textile manufacturers located in Samut Prakan Province, Thailand. The wastewater contained three different synthetic reactive dyes: Novacron NAVY EC-R (NEC-R), Novacron RUBY S3B (NR-S3B) and Novacron Super Black G (NSB-G), as they were used during the decolourisation process. The information for the wastewater was analysed by the Environmental Research Institute, Chulalongkorn University, and was compared to the industrial effluent standard from the Ministry of Industry in Thailand [14], as shown in Table 1.

The chemicals used in the ligninolytic enzyme activity were purchased from Sigma (St. Louis, MO, USA). The culture media were procured from either Fisher Scientific (Singapore) and Himedia (Mumbai, India).

### 2.2. Microorganisms

A total of 16 isolates of white-rot fungi were obtained from our previous study [7] (Table S1). Culture collections were preserved at the Department of Microbiology, Faculty of Science, Srinakharinwirot University, Thailand. They were cultured on potato dextrose agar at 30 °C. These were *Amauroderma subresinosum* NP17-12 (OM996018), *Coriolopsis aspera* NP17-02 (MK589268), NP17-08 (MK589269), *C. retropicta* PW17-134 (MK589270), *Dentipellis parmastoi* PW17-136 (MK589290), *Ganoderma fornicatum* PW17-145 (MK589271), *G. lingzhi* PW17-43 (MK589272), *G. mastoporum* PW17-06 (MK589273), PW17-154 (MK589275), *Microporus vernicipes* PW17-173 (MK589280), *M. xanthopus* PP17-17 (MK589281), PP17-20 (MK589282), *Pseudolagarobasidium* sp. PP17-33 (MK589289), *Trametes elegans* PP17-06 (MK589285), *T. hirsuta* PW17-41 (MK589286) and *T. sanguinea* PP17-18 (MK589287). The GenBank accession numbers of internal transcribed spacer sequences are represented in parentheses.

### 2.3. Screening for Decolourisation of Textile Dyes by White-Rot Fungi

Decolourisation experiments were carried out in a 250 mL Erlenmeyer flask. Five plugs (8.75 mm in diameter from a seven-day-old agar culture) of each fungal isolate were cultivated in 50 mL of liquid basal medium containing 10 g L^−1^ glucose, 1 g L^−1^ peptone, 1 g L^−1^ KH_2_PO_4_, 0.5 g L^−1^ MgSO_4_.7H_2_O, 0.05 g L^−1^ Na_2_HPO_4_, 0.01 g L^−1^ CaCl_2_, 0.01 g L^−1^ FeSO_4_.7H_2_O, 0.001 g L^−1^ MnSO_4_.4H_2_O, 0.001 g L^−1^ ZnSO_4_.4H_2_O, 0.002 g L^−1^ CuSO_4_.5H_2_O and 4% (*v/v*) (or 2,475 ADMI) of textile dyes (pH 5.5) at 150 rpm and 30 °C for seven days. The fungal pellets were removed using Whatman No. 1 filter paper. The supernatant was then filtered through a membrane filter measuring 0.45 µm before the dye concentration was measured in ADMI units using a spectrophotometer (HACH DR3900, Düsseldorf, Germany) [15]. The percentage of dye decolourisation was calculated according to the formula described by Maniyam et al. [16].

### 2.4. Fungal Cell Immobilisation on Different Supports

Four different supports, namely a nylon sponge (commercially available sponge), loofah, parchment coffee (lignocellulose waste of green coffee bean’s outer layer) and cascara coffee, were used for fungal cell immobilisation. A nylon sponge was soaked in distilled water overnight, boiled for 10 min and washed three times with distilled water before use [17]. The loofah, parchment coffee and cascara coffee were washed with distilled water before drying. The nylon sponge and loofah were cut into 1 cm^3^ cubes, dried at 50 °C for 24 h and kept in a desiccator.

Three mycelium plugs of the selected fungus, *T. hirsuta* PW17-41, were grown in a 250 mL Erlenmeyer flask containing 50 mL of potato dextrose broth and supporters (15 cubes for nylon sponge and loofah, 1 g for parchment coffee and 2 g for cascara coffee) on an orbital shaker at 120 rpm and 30 °C for five days. Then, the supporters containing fungal mycelium were taken out and washed twice in sterile distilled water. They were dried at 50 °C for 48 h and kept in a desiccator before measuring the cell dry weight. All treatments were performed in triplicate.

### 2.5. Effects of Carbon, Nitrogen, pH and Agitation Speed on Textile Dye Decolourisation by Immobilisation of T. hirsuta PW17-41

The experiments were performed in 250 mL Erlenmeyer flasks containing 50 mL of basal medium. The effects of carbon and nitrogen sources on the textile dye decolourisation were observed. Five immobilised fungal mycelia on nylon sponges were grown in the medium using different carbon sources (fructose, galactose, lactose, glucose, palm sugar, soluble starch, sucrose). A supplement of 4% (*v/v*) textile dyes was added and the pH was adjusted to 5.5. The culture was grown on an orbital shaker at 150 rpm and 30 °C for 48 h. The absorbance levels in ADMI values were measured and calculated for the percentages of decolourisation.

The effects of the nitrogen source (peptone, NaNO_3_, NH_4_NO_3_, (NH_4_)_2_SO_4_, CH_4_N_2_O or yeast extract) were observed. The carbon source was selected from the previous experiment containing the highest percentage of decolourisation. The supplement of 4% (*v/v*) textile dyes (or 2475 ADMI) was added and grown in the same condition as above. The initial pH levels (4, 5, 6, 7, 8, 9 and 10) were analysed using the carbon and nitrogen sources containing the highest percentages of decolourisation. Finally, the effects of various agitation speeds (50, 100 or 150 rpm) were also observed. All measurements were performed in triplicate.

### 2.6. Adsorption of Textile Dyes by Dead and Living Biomass on Support

The adsorption of textile dyes by dead cells of immobilised *T. hirsuta* PW17-41 was evaluated under optimal conditions containing 4% (*v/v*) textile dyes (or 2475 ADMI), compared with live cells of immobilised *T. hirsuta* PW17-41, fungal mycelium and nylon sponges. The percentage of adsorption was calculated according to the formula below:

% Adsorption = (ADMI_0_ − ADMI_1_)/ADMI_0_ × 100

where ADMI_0_ is the absorbance value of the original dye concentration and ADMI_1_ is the absorbance value of the dye concentration after being treated with fungi.

### 2.7. Effect of the Initial Textile Dye Concentrations on Decolourisation by Immobilisation of T. hirsuta PW17-41

The initial concentrations of textile dyes (4, 8, 17, 25, 33, 42, 50, 66, 83 and 100% (*v/v*)) were evaluated by using five pieces of immobilised *T. hirsuta* PW17-41 in a 250 mL Erlenmeyer flask containing 50 mL of optimal medium. The agitation speed was 100 rpm at 30 °C. The percentages of decolourisation were measured at zero, two, four, six and eight days. The experiments were accomplished in triplicate.

### 2.8. Time Course Study of the Relationship between Textile Dye Decolourisation and Enzyme Activities

The decolourisation of textile dyes by immobilised *T. hirsuta* PW17-41 was measured using a time course study. The experiments were performed in 250 mL Erlenmeyer flasks containing 50 mL of optimal medium. The reaction mixture contained 5 pieces of fungal mycelium immobilised on nylon sponges, 10 g L^−1^ palm sugar, 1 g L^−1^ NH_4_NO_3_, 1 g L^−1^ KH_2_PO_4_, 0.5 g L^−1^ MgSO_4_.7H_2_O, 0.05 g L^−1^ Na_2_HPO_4_, 0.01 g L^−1^ CaCl_2_, 0.01 g L^−1^ FeSO_4_.7H_2_O, 0.001 g L^−1^ MnSO_4_.4H_2_O, 0.001 g L^−1^ ZnSO_4_.4H_2_O, 0.002 g L^−1^ CuSO_4_.5H_2_O and 33% (*v/v*) (or 21,200 ADMI) of textile dyes (pH 5) at 100 rpm and 30 °C for 20 days. The percentages of decolourisation and enzyme activity (laccase, MnP and LiP), pH values and cell dry weights were measured every two days for 20 days.

### 2.9. Repeated Batch Experiments

Five pieces of immobilised *T. hirsuta* PW17-41 were cultured in 250 mL Erlenmeyer flasks containing 50 mL of optimal medium with 4% (*v/v*) textile dyes. After two days, the pieces of immobilised cells were transferred to a new medium with the same concentrations of textile dyes and repeated 12 times. The dye decolourisation and ligninolytic enzyme production in each batch were determined. The efficiency of total decolourisation in repeated batches was calculated followed this equation [18]:

$$\%\;\mathrm{Total}\;\mathrm{decolourisation}\;\mathrm{efficiency}=\Sigma \;\left(C_{0}-{\;C}_{t}\right)/{\Sigma \;C}_{0}\times 100$$

where C_0_ is the initial ADMI value of each cycle and C_t_ is the ADMI value after a two day incubation period.

### 2.10. Laccase, MnP and LiP Production by T. hirsuta PW17-41

Ligninolytic enzymes, laccase, MnP and LiP were analysed. Laccase activity was determined using the method described by Machado and Matheus [19] with modifications. The reaction mixture contained 600 µL of 0.5 mM ABTS in 100 mM sodium acetate buffer (pH 4) and 600 µL of the sample. After incubation at 60 °C for 1 min, the absorbance of the mixture was measured at 420 nm (*ε* = 36,000 M^−1^ cm^−1^). The MnP activity was determined by monitoring the oxidation of 2,6-dimethoxyphenol (DMP) according to the description of Thamvithayakorn et al. [7]. The reaction mixture contained 120 µL of 10 mM DMP, 240 µL of 250 mM sodium tartrate buffer (pH 4), 120 µL of 10 mM MnSO_4_, 120 µL of 4 mM H_2_O_2_ and 600 µL of sample. The mixture was incubated at 60 °C for one minute and the absorbance of the mixture was measured at 469 nm (*ε* = 10,000 M^−1^ cm^−1^) [20,21]. Lignin peroxidase activity was determined using the method described by Tien and Kirk [22] with modifications. The reaction mixture contained 240 µL of 20 mM veratryl alcohol, 240 µL of 250 mM sodium tartrate buffer (pH 4), 120 µL of 4 mM H_2_O_2_ and 600 µL of sample. After incubation at 60 °C for one minute, the mixture was measured and the absorbance was at 310 nm (*ε* = 9300 M^−1^ cm^−1^). One unit (U) of ligninolytic enzyme activity is the amount of enzyme transforming 1 µmol of substrate per minute.

### 2.11. Total Protein Determination, MnP Purification, Sodium Dodecyl Sulfate–Polyacrylamide Gel Electrophoresis (SDS-PAGE) and Native-PAGE Analysis

The total protein was measured using the Bradford protein assay. The standard curves of bovine serum albumin were measured at different concentrations of 0, 20, 40, 60, 80 and 100 µg mL^−1^ [23]. Two hundred microliters of crude enzyme was mixed with 1 mL of Bradford’s reagent. After agitation, the absorbance was measured at 595 nm.

For enzyme production, five mycelial plugs of *T. hirsuta* PW17-41 were cultured in an optimal medium without textile dyes at 100 rpm and 30 °C for seven days. The crude MnP was obtained by filtration of fungal culture using Whatman No. 1 filter paper. The protein was precipitated using (NH_4_)_2_SO_4_ of 80% saturation overnight. Then, the protein precipitate was collected by centrifugation and was dissolved in 30 mL of sodium phosphate buffer (0.05 mM, pH 6.0). The solution was placed into a dialysis bag, cut off at 8 kDa and dialysed in the same buffer overnight by changing the buffer twice. The crude MnP was measured for enzyme activity and enzyme specificity.

The molecular mass of MnP was measured using the SDS-PAGE method, according to the protocol described by Laemmli [24]. The sample was run in polyacrylamide gel containing 5% (*w/v*) stacking gel and 12% (*w/v*) separating gel. After gel staining with Coomassie Brilliant Blue R-250 for 30 min, the molecular mass of MnP was identified with the standard protein markers. A Native-PAGE analysis was performed on a 12% (*w/v*) separating gel without SDS. Then, the gel was incubated with a 50 mM sodium tartrate buffer (pH 4) containing 0.4 mM H_2_O_2_, 1 mM MnSO_4_ and 1 mM DMP.

### 2.12. UV–Visible and Fourier Transform Infrared Spectroscopy (FTIR) Analysis for Biodegradation of Textile Dyes by T. hirsuta PW17-41

The decolourisation manner of textile dyes by immobilised *T. hirsuta* PW17-41 was investigated by collecting the treated textile dyes at zero, four, 12 and 20 days under the same conditions as for the treatment described in the time course study. The spectrophotometric scanning was observed in the range of 320–700 nm (HACH DR3900, Düsseldorf, Germany). The infrared spectra of dyes on day zero and 12 were analysed using an FT-IR Spectrometer INVENIO (Bruker, Billerica, MA, USA). The treated wastewater was extracted by ethyl acetate and concentrated by a rotary evaporator. Then, the FTIR spectra of textile dyes and their degraded products were analysed [25].

### 2.13. Statistical Analysis

All data were analysed in triplicate and presented as the means ± standard deviation. The standard deviation and significance level were calculated using SPSS software. The one-way analysis of variance (ANOVA) with Tukey post hoc test was conducted to obtain the statistical significance between mean values, with *p* values < 0.05.

## 3. Results and Discussion

### 3.1. Screening for Decolourisation of Textile Dyes by White-Rot Fungi

**Figure 1:**
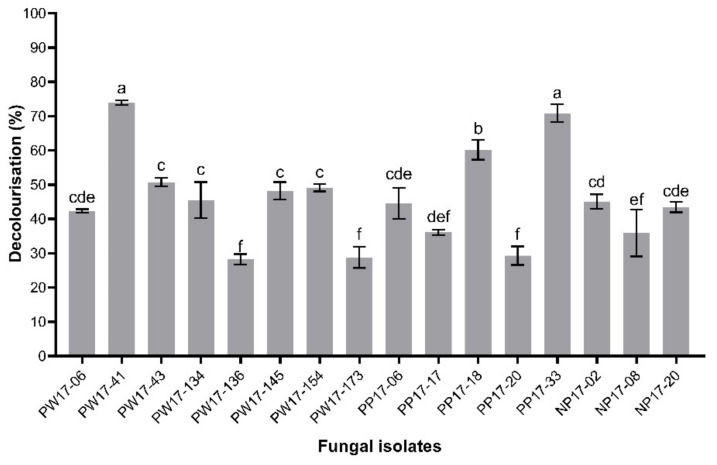
Ability of different white-rot fungi for textile dye decolourisation under submerged conditions. Error bars indicate the standard deviation, while letters a–f indicate statistically significant differences between groups analysed via one-way ANOVA (*n* = 3, *p* < 0.05).

Large amounts of dyestuffs are used in the dyeing process and contain a wide range of structurally diverse dyes. The removal of these dyes from wastewater is an essential process before being discharged into the environment. Ligninolytic enzymes secreted by white-rot fungi have been reported to be exploited for the decolourisation of different synthetic dyes. The screening of new fungal strains having a strong ability to decolourise textile dye is, therefore, still of interest. The dyes used in the present study were a mixture of three different synthetic dyes, NEC-R, NR-S3B and NSB-G, which were obtained from the textile industry after the dyeing process. The standard method of dye determination from dye mixture or wastewater was evaluated via the American Dye Manufacturers’ Institute (ADMI) standard value, which was obtained from the spectrophotometric scanning in the range of 320–700 nm of dye samples. This method covered all wavelengths of visible colours suitable for dye mixture, and the well-defined peaks did not show in the spectrophotometer [26]. Therefore, the decolourisation of textile dyes in the present study was determined from the ADMI values. The industrial effluent standard of the colour discharging from a manufacturer should be lower than 300 ADMI [14]. Our 16 fungal strains belonged to 7 genera (*Amauroderma*, *Coriolopsis*, *Dentipellis*, *Ganoderma*, *Microporus*, *Pseudolagarobasidium*, *Trametes*) and 13 species exhibited different percentages of decolourisation efficiency varying from 28.3% to 74.0% at an initial concentration of 4% (*v/v*) of textile dyes (or 2475 ADMI) within seven days. Similarly, different fungi had different dye decolourisation abilities. *Trametes hirsuta* PW17-41 showed the highest decolourisation rate (74.0%), followed by *Pseudolagarobasidium* sp. PP17-33 (70.9%) and *T. sanguinea* (PP17-18) (60.2%), which had final ADMI values of 644, 721 and 986, respectively (Figure 1 and Table S2). Remarkably, all fungal isolates could grow in real textile wastewater containing a high level of salinity (70,000 mg L^−1^) and heavy metal ions (Table 1). Due to the synthetic dyes used in the present study being reactive dyes, they required NaCl to neutralise the fibre surface charge to increase the capacity for dye adsorption. The fungus *T. hirsuta* has been reported as a potential species for several kinds of dye decolourisation, including Indigo Carmine, Phenol Red, Bromophenol Blue, Methyl Orange and Poly R-478 [11,27,28,29]. Recently, members of this genus have proved capable of efficient decolourisation of wastewater from the textile industry. Isolated *T. sanguinea* H-1 was able to decolourise synthetic dyes such as Congo Red, Malachite Green and Methylene Blue [30].

Furthermore, ligninolytic enzymes from different *T*. *sanguinea* strains exhibited good potential for applications in industrial dye bioremediation, detoxification and decolourisation processes [31,32,33]. For *Pseudolagarobasidium* sp., only *P. acaciicola* AGST3 showed 11–96% decolourisation efficiency when treated with different dyes after 24 h of incubation [34]. However, the present study revealed that many white-rot fungal strains from our collections could be considered good candidates for dye decolourisation in real textile wastewater.

### 3.2. Fungal Cell Immobilisation on Different Supports

**Figure 2:**
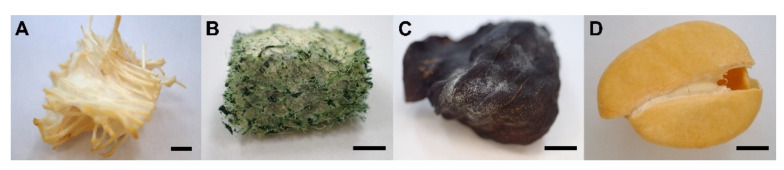
Immobilisation of *Trametes hirsuta* PW17-41 on different solid supports: (**A**) nylon sponge; (**B**) loofah; (**C**) cascara coffee; (**D**) parchment coffee. Bars = 0.1 cm.

Four different supports, i.e., nylon sponge, loofah, cascara coffee and parchment coffee, were investigated for fungal immobilisation. *Trametes hirsuta* PW17-41 was selected to immobilise the fungal cells on different carriers and the results revealed that nylon sponge was the most suitable support for fungal immobilisation (Table S3). The biomass of *T. hirsuta* PW17-41 on nylon sponges (0.375 ± 0.013 g) was higher than that of the loofahs (0.255 ± 0.021 g). However, the fungus grew well, attaching to both supports, and the liquid phase remained clear during the whole cultivation period, whereas mycelial growth with parchment coffee (0.099 ± 0.007 g) and cascara coffee (0.001 ± 0.000 g) materials contained only a few fungal mycelia (Figure 2). This might be because their surface characteristics were smooth, making it difficult for fungal growth and attachment under submerged conditions. Nylon sponges and loofahs have greater potential for fungal immobilisation due to a greater number of pores and rough surfaces, which allows the fungal mycelium to penetrate the pores, engage in rapid mycelium development and cover the surface area of the materials (Figure 2A,B). In addition, all supports were tested for dye adsorption and the results showed no adsorption of all the immobilisation materials tested (data not shown). The high capacity levels for dye adsorption on different solid supports have been widely studied. Rodríguez-Couto et al. [28] reported that immobilised *T. hirsuta* BT2566 on stainless steel sponge in a bioreactor could degrade the textile dye Indigo Carmine up to 96% within one day, which is a much higher decolourisation percentage than the culture filtrate (74% in 2 days). Przystás et al. [35] studied the efficiency of different solid supports for fungal immobilisation (*Pleurotus ostreatus* BWPH, *Gleophyllum odoratum* Dca and *Polyporus picipes* RWP17) and demonstrated that the brush and washer were good carriers, as they have high levels of biomass immobilisation. The immobilisation of biomass improved the dye removal.

### 3.3. Effects of Carbon, Nitrogen, pH and Agitation Speed on Textile Dye Decolourisation by Immobilisation of T. hirsuta PW17-41

To explore the optimal dye decolourisation conditions using immobilised *T. hirsuta* PW17-41, the effects of carbon and nitrogen sources, pH levels and agitation speeds on the textile dye decolourisation percentage were determined.

#### 3.3.1. Effect of Carbon Source

**Figure 3:**
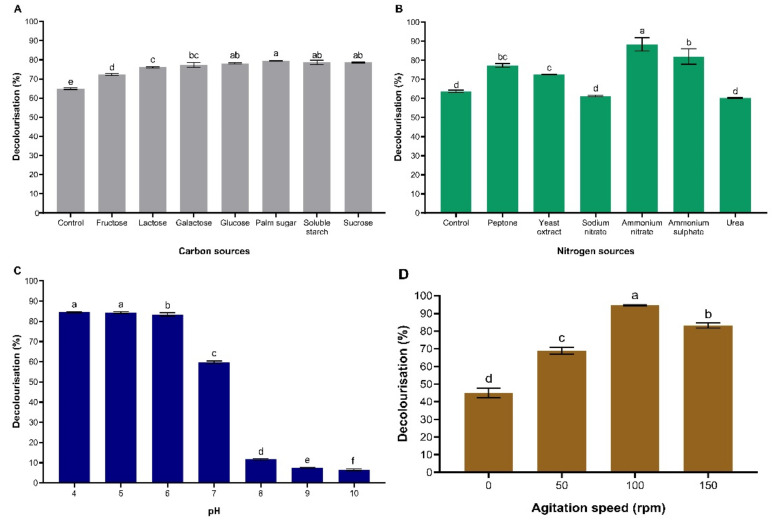
Effects of carbon (**A**), nitrogen (**B**), pH (**C**), and agitation speed (**D**) on decolourisation of textile dyes by immobilisation of *T. hirsuta* PW17-41 at 4% (*v/v*) concentration after 48 h. Error bars indicate the standard deviation and letters a–f indicate statistically significant differences between groups analysed via one-way ANOVA (*n* = 3, *p* < 0.05).

The dye decolourisation efficiency caused by immobilisation of *T. hirsuta* PW17-41 was significantly influenced by medium composition. The effects of carbon sources on textile dye decolourisation efficiency were high in palm sugar (79.47%), soluble starch (79.28%) and sucrose (78.61%) (Figure 3A and Table S4). The explanation of the higher decolourisation using palm sugar as a carbon source could be the nutritional contribution of the sources, which resulted in high decolourisation of textile dyes. Palm sugar is a natural sweetener that contains several nutrients. Maryani et al. [36] analysed the contents of palm sugar and found that it consisted of 89.94% sucrose, 3.61% glucose and 3.50% fructose. Because the major content of palm sugar is sucrose, the result of dye decolourisation using sucrose alone as a carbon source was not significantly different from palm sugar. The external carbon source was necessary for fungi to degrade polycyclic aromatic hydrocarbons, including synthetic dyes. The study by Nilsson et al. [37] revealed that *T. versicolor* showed high decolourisation rates for both Reactive Blue 4 and Reactive Red 2 when adding glucose as a carbon source. Therefore, palm sugar was selected for use as a carbon source in the next experiment.

#### 3.3.2. Effect of Nitrogen Source

The effects of nitrogen sources are shown in Figure 3B and Table S3. Compared to the inorganic nitrogen sources of NaNO_3_, NH_4_NO_3_ and (NH_4_)_2_SO_4_, the decolourisation efficiency was significantly improved for cultures supplemented with organic nitrogen from yeast extract, urea and peptone. The cultures with NH_4_NO_3_ showed the highest percentage of dye decolourisation (87.81%) among the other nitrogen sources. Ammonium nitrate might be an electron acceptor preferentially consumed by *T. hirsuta* PW17-41. Previous studies have also reported that NH_4_NO_3_-supplemented culture leads to enhanced decolourisation efficiency of *Trametes versicolor* [38]. Mikiashvili et al. [39] reported that the utilisation of NH_4_NO_3_ accelerated the secretion of MnP up to 44-fold, while medium supplementation with NaNO_3_, NH_4_H_2_PO_4_ and (NH_4_)_2_SO_4_ inhibited this enzyme accumulation by *T. versicolor* strain 775. Asgher [40] studied the laccase production from *T. versicolor* IBL-04 and found that carbon and nitrogen sources could enhance the ligninolytic enzyme production involved in dye removal. The lowest decolourisation for the culture was supplemented with urea (60.30%), which was lower than the control (63.79%) without a nitrogen source. This might be the effect of ligninolytic enzyme inhibition. Several studies revealed that the concentration and type of nitrogen source had effects on ligninolytic enzyme production and regulation by white-rot fungi, especially in basidiomycetes. These factors were essential for dye decolourisation by fungi [41]. The type and number of ligninolytic enzymes from most white-rot fungi depended on the nitrogen source in the fungal culture. In addition, Moldes et al. [42] demonstrated that the nitrogen source influenced the proportion of laccase isoenzyme production by *T. versicolor*, which played an important role in dye decolourisation.

#### 3.3.3. Effect of pH

The effect of pH on textile dye decolourisation was evaluated by adjusting the initial pH of the wastewater from 4 to 10. The decolourisation of textile dye by immobilised *T. hirsuta* PW17-41 showed high percentages of decolourisation at pH 4 to 6, ranging from 83.42 to 84.57%. The highest percentages of decolourisation were observed at pH 4 (84.57%) and 5 (84.40%) (Figure 3C and Table S3). The decolourisation decreased when the pH was increased. The results indicated that better decolourisation of textile dyes was achieved under acidic conditions. Many studies report that the suitable pH range for dye decolourisation by microorganisms is 5.5–7 [43,44]. However, the report by Pearce et al. [45] demonstrated that some white-rot fungi had an optimum pH at neutral or at a slightly alkaline pH for dye decolourisation. This might be because the pH affected the membrane transportation of dye molecules passing through the cell membrane, which limited the dye decolourisation [46]. Therefore, pH 5 was selected in the present study.

#### 3.3.4. Effects of Agitation Speed

In this study, the effects of the agitation speed were investigated at 0, 50, 100 and 150 rpm by culture in an optimal medium using palm sugar and NH_4_NO_3_ as carbon and nitrogen sources, respectively, at pH 5 and 30 °C. The immobilised *T. hirsuta* PW17-41 showed the highest decolourisation when the agitation speed was 100 rpm (94.60%), followed by 150 rpm (83.18%) and 50 rpm (68.80%) (Figure 3D and Table S4). The dye decolourisation potential of the culture was the lowest (45.01%) at 0 rpm (static condition). The agitation helped to increase the oxygen transfer between cells and culture medium and led to an increase in fungal biomass. However, the decolourisation activity was suppressed when the agitation speed was increased to 150 rpm. This might be due to the high speed of agitation and deactivation of the enzymes responsible for decolourisation. Similar results have been reported by Aksu and Dönmez [47], where at an agitation speed of 100 rpm, the fungus *T. versicolor* was able to decolourise 88% of Remazol Blue reactive dye within 4 days of incubation.

### 3.4. Effects of the Initial Textile Dye Concentrations on Decolourisation by Immobilised T. hirsuta PW17-41

**Figure 4:**
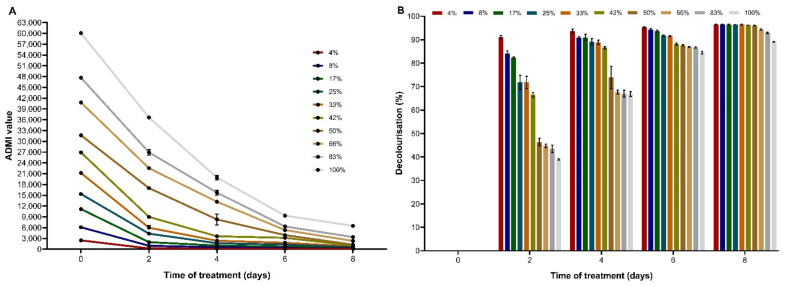
Effect of the initial textile dye concentration (4%, 8%, 17%, 25%, 33%, 42%, 50%, 66%, 83% and 100% (*v/v*)) on decolourisation by immobilisation of *T. hirsuta* PW17-41 after 8 days incubation: (**A**) ADMI values of decolourisation process; (**B**) % decolourisation at different dye concentrations. Error bars indicate standard deviation.

The decolourisation by immobilised *T. hirsuta* PW17-41 was studied at different initial textile dye concentrations varying from 4 to 100% (*v/v*). The decolourisation rate of immobilised fungal cells was decreased when the dye concentration was increased (Figure 4 and Table S5). The initial dye concentration of 4% exhibited the lowest ADMI value of 217 (91.13% decolourisation) within 2 days, which decreased to 85 ADMI (96.54% decolourisation) after 8 days. The increasing dye concentration of 8 to 100% showed 94.38 to 89.17% decolourisation after treatment. Several reports have been studied on the initial dye concentration. Similarly, Chen and Ting [48] reported that colour removal of Methyl Violet, Crystal Violet and Malachite Green appeared to be better at lower initial dye concentrations by *Coriolopsis* sp. Khan and Fulekar [49] simulated wastewater using *Aspergillus bombycis* for dye decolourisation of a sulfonated textile dye, Reactive Red 31, and demonstrated that after incubation for 12 h, dye concentration was decreased from 99.76 to 91.06% as the concentration increased from 5 to 25 mg L^−1^. As they described, the efficiency of the dye decolourisation depended on the initial concentration. The lower concentrations of the dyes were easily decolourised, while the high concentrations decreased the decolourisation. This might be because of the toxicity of dyes to fungal cells.

### 3.5. Adsorption Capacity of Immobilised T. hirsuta PW17-41

**Figure 5:**
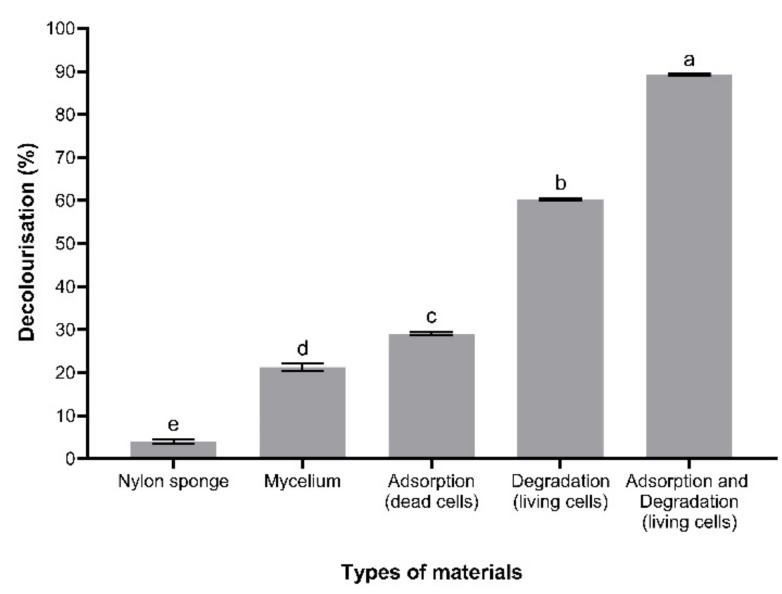
Adsorption capacity of nylon sponge, fungal mycelium, dead cells of immobilised *T. hirsuta* PW17-41 and living cells of immobilised *T. hirsuta* PW17-41 for textile dye decolourisation under optimal conditions at 4% (*v/v*) initial dye concentration for 2 days at 100 rpm and 30 °C. Error bars indicate standard deviation and letters a–e indicate statistically significant differences between groups analysed via one-way ANOVA (*n* = 3, *p* < 0.05).

The dead cells of immobilised *T. hirsuta* PW17-41 were observed for their adsorption capacity under optimal conditions, and the results exhibited 28.98% decolourisation (Figure 5 and Table S6). The living immobilised fungal cells showed 89.21% decolourisation, which indicated that biodegradation (60.23%) was a main mechanism of textile dye decolourisation in the present study. The fungal mycelium without immobilisation showed only 21.28% decolourisation, while the nylon sponge could adsorb only 4.04% of textile dyes. Due to the fungal cell wall consisting of polysaccharides, protein and lipids, the molecules of dyes can bind to the active groups on the cell walls via van der Waals forces or chemical mechanisms [50]. Przystas et al. [51] used the dead biomass of the fungus *Pleurotus ostreatus* to adsorb a dye mixture (Triphenylmethane Brilliant Green and Azo Evans Blue), resulting in 37% decolourisation. Living biomass could remove dyes at up to 79.2% under a shaking condition, while the static condition showed 51.9% removal. However, the shaking condition improved the efficacy and rate of dye removal.

### 3.6. Time Course Study of the Relationship between Textile Dye Decolourisation and Enzyme Activities

**Figure 6:**
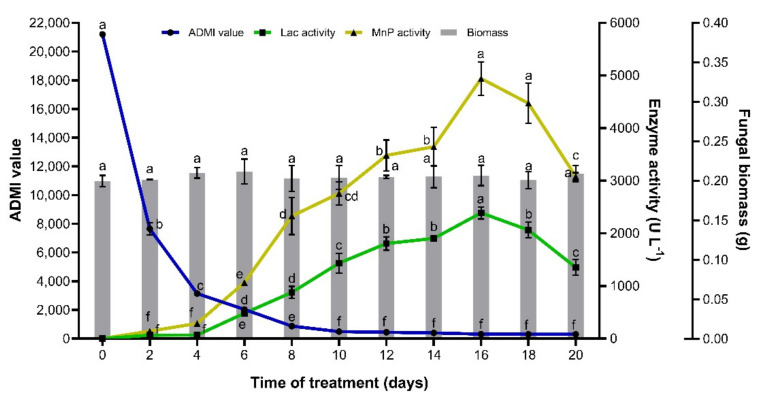
A time course study of textile dye decolourisation and MnP and laccase production by immobilised *T. hirsuta* PW17-41 under a submerged cultivation condition using 33% (*v/v*) dye concentration (or 21,200 ADMI) at 30 °C and 100 rpm for 20 days. Error bars indicate standard deviation and letters a–g indicate statistically significant differences between groups analysed via one-way ANOVA (*n* = 3, *p* < 0.05).

Optical conditions previously observed were used to study dye decolourisation by immobilised *T. hirsuta* PW17-41. The decolourisation and ligninolytic enzyme production were conducted with 33% concentration (or 21,200 ADMI) of textile dyes for 20 days. The results revealed that the biomass of fungal cells was stable and that pH decreased in the first seven days and then stabilised (Figure 6 and Table S7). The efficiency of dye decolourisation was increased and reached a maximum amount of 98.59% decoulrisation after 20 days of incubation (Figure 6). During the initial stage of the reaction on days 1–8, the decolourisation percentage increased significantly and tended to be more stable at later time points. On day 4, approximately 85.18% of textile dyes were removed. The ADMI value decreased from 21,200 to 493 on day 10 (97.67%) and slightly decreased further to 300 ADMI on day 20 (98.59%), which passed the industrial effluent standard. This exhibited an approximately 71-fold decrease in textile dye decolourisation. The colour of textile dyes was changed from navy to dark red after 4 days of treatment and then to pink at day 12 before changing to light pink on day 20 (Figure S1). Due to the textile dyes being a mixture of three different dyes, NEC-R, NR-S3B and NSB-G, it was possible that NEC-R and NSB-G were degraded first, followed by NR-S3B. However, at 4% (*v/v*) of the initial dye concentration (or 2475 ADMI), the colour changed from navy blue to very pale pink after treatment with immobilised *T. hirsuta* PW17-41 for 8 days and yielded a final ADMI value of 85.

For ligninolytic enzyme production, only MnP and laccase were detected in the system after 2 days, while LiP could not be observed. The result supported our previous study by Thamvithayakorn et al. [7], which examined ligninolytic enzymes using oil palm decanter cake as a substrate, and found that *T. hirsuta* PW17-41 could produce only MnP and laccase. Vasina et al. [52] studied the secretome analysis of the *T. hirsuta* strain 072 cultivated in synthetic media and a lignocellulosic substrate using MALDI-TOF/TOF MS analysis, but could not detect LiP during the ligninolytic enzyme production. This fungus produced high levels of laccase and peroxidase in various media, especially when adding the lignocellulosic materials. In the present study, MnP was a major ligninolytic enzyme in the textile dye degradation process, which was slightly increased and exhibited the most activity after 16 days of treatment (4942 U L^−1^), while laccase (2389 U L^−1^) showed less activity than MnP during the decolourisation process. Therefore, the main mechanism of textile dye decolourisation by immobilised *T. hirsuta* PW17-41 was the degradation by MnP and laccase. Several studies have shown that ligninolytic enzymes are efficient in dye decolourisation [53]. The presence of the new MnP (named MnP TP55) from *Trametes pubescens* strain i8 during textile dye decolourisation was mainly produced by the fungus [54]. The MnP TP55 was then used to degrade several types of dyes, for example anthraquinone dyes, triphenylmethane dye, indigo dye, azo dyes, polymeric dye, and acid dye, reaching decolourisation rates of 42%, 46%, 50%, 64%, 66% and 76%, respectively, within 24 h at an initial dye concentration of 50 μM. In addition, *Irpex lacteus*, *Bjerkandera adusta*, *T. versicolor* and *P. chrysosporium* are white-rot fungi that are widely recognised as excellent biodegradable agents with a high capability and efficiency for producing ligninolytic enzymes for degrading a wide array of synthetic dyes. [55,56]. Nonetheless, some studies have reported [6] that mainly laccases and MnP are produced by the genus *Trametes*; only a few reports have discovered that *T. hirsuta* could produce a high amount of MnP. Rodríguez-Couto and Sanromán [57] employed cultures of *T. hirsuta* immobilised on coconut flesh to study the decolourisation of the industrial dye, Lissamine Green B. They found that extracellular laccase secreted by the fungus mainly reacted with decolourised dye. Karimi et al. [58] reported that the synthetic dye, Methylene Blue, was totally decolourised by *P. chrysosporium* immobilised on the mineral Kissiris due to the action of MnP produced by the fungus. Nuryana et al. [59] studied MnP and laccase production from *T. hirsuta* by supplementing it with aromatic compounds and found that the fungus secreted a high level of laccase but a low level of MnP when cultured in glucose yeast peptone broth under submerged conditions. Jovic et al. [60] observed two isolates of *T. hirsuta* F13 and *Stereum gausapatum* F28 for lignin removal from beech wood sawdust by ligninolytic enzymes. The results revealed that *T. hirsuta* F13 produced high amounts of laccase, while *S. gausapatum* F28 produced high amounts of MnP. In total, 33.84% and 28.8% of the lignin content of beech wood sawdust were removed by *T. hirsuta* F13 and *S. gausapatum* F28, respectively. In the present study, it was demonstrated that under submerged cultivation conditions, higher decolourisation efficiency was achieved with the immobilised *T. hirsuta* PW17-41. This is important for the textile industry, especially for further applications in the dye discolourisation of wastewater.

### 3.7. Manganese Peroxidase Production by Trametes hirsuta PW17-41

**Figure 7:**
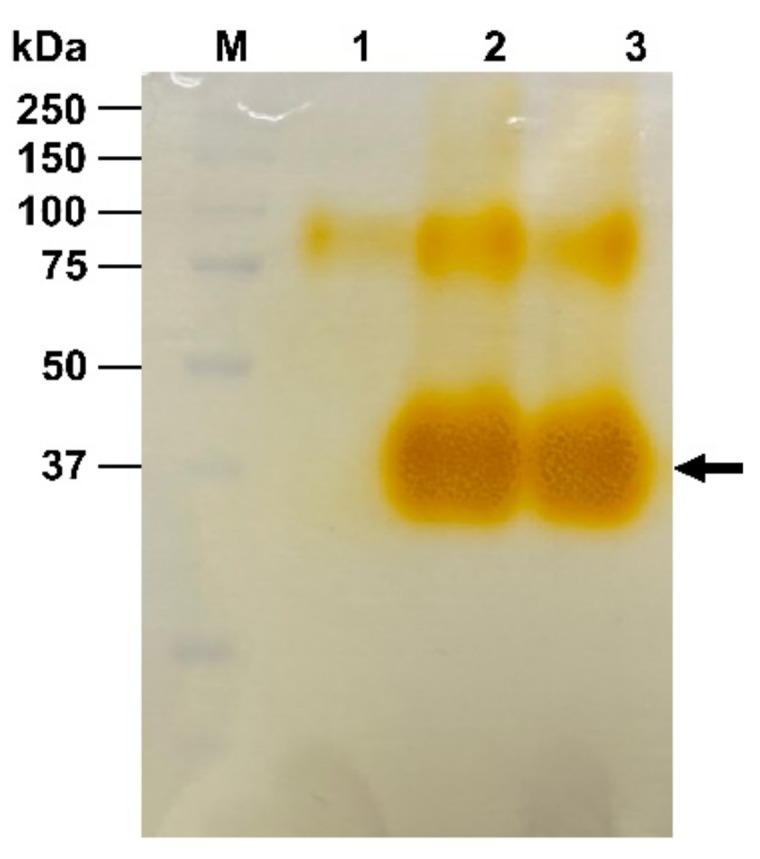
Native-PAGE image of MnP from *Trametes hirsuta* PW17-41 after reacting with ABTS and H_2_O_2_. Lane M = molecular weight marker (kDa); lane 1 = culture broth; lane 2 = (NH_4_)_2_SO_4_ precipitation; lane 3 = dialysis.

The liquid culture obtained from *T. hirsuta* PW17-41 was purified to homogeneity using ammonium sulfate precipitation followed by dialysis. The results of MnP purification at different steps are summarised in Table S8. The crude filtrate subjected to dialysis showed specific activity of 404.247 U mg^−1^ with 4.852-fold purification. The molecular weight of MnP was analysed by SDS-PAGE and was confirmed by Native-PAGE, which reacted with the substrate. The active band exhibited an orange colour after being incubated with 1 mM DMP, 1 mM MnSO_4_ and 0.4 mM H_2_O_2_. The molecular mass of MnP was found to be approximately 45 kDa (Figure 7), resembling the molecular mass of most fungal MnPs ranging from 38 to 62.5 kDa [61,62]. *Trametes* sp. 48424 and *Rhizoctonia* sp. SYBC-M3 produced MnPs with molecular mass as 49 kDa [63] and 40.4 kDa [64], respectively. Moreover, white-rot fungi usually contain multiple MnPs encoding genes as a gene family. Therefore, MnP has more than one isoenzyme, such as *T. pubescens* FBCC735 (10 isoenzymes) and *P. ostreatus* PODs (5 isoenzymes) [54].

### 3.8. Repeated Batcingh of Textile Dye Decolourisation by Reusing the Immobilised T. hirsuta PW17-41

Twelve repeated cycles were carried out by reusing the immobilised *T. hirsuta* PW17-41 in new dyes every 2 days at the same initial dye concentration of 2475 ADMI. The decolourisation efficiency was still high, varying from 89.28 to 95.77% until the last cycle (Figure S2). Notably, MnP and laccase activities increased from the first to the last cycle by approximately 7.6-fold and 4.8-fold, respectively. The highest activity levels of Mn (7151 U L^−1^) and laccase (2220 U L^−1^) were observed on the sixth cycle before slightly decreasing to 4796 U L^−1^ for MnP and 1198 U L^−1^ for laccase (Table S9). The percentages of decolourisation were related to the enzyme activities. Interestingly, all cycles exhibited final ADMI values lower than 300, which passed the industrial effluent standard. The total decolourisation efficiency was 93.61%. Our results demonstrated that the immobilised fungal mycelia could maintain high decolourisation efficiency over long-term operation. Yesilada et al. [65] reported high and stable decolourisation activity levels of Astrazone Blue dye using white-rot fungus *Funalia trogii* pellets during the repeated batch, and the percentage of decolourisation remained high after five cycles of operation. Krastanov et al. [66] observed the stability of repeatedly used *T. versicolor* pellets to decolourise the industrial dyes Orange II and Reactive Blue 4 for five cycles, and found that there was a rapid loss of dye decolourisation activity shortly after the third cycle. In the case of Reactive Violet 12, it could be reused for seven cycles. However, the types and initial concentrations of dyes were important factors for repeated use in the decolourisation process. The most significant advantage of immobilised fungal mycelia is the possibility of designing continuous processes in further application.

### 3.9. UV–Visible and Fourier Transform Infrared Spectroscopy (FTIR) Analysis for Biodegradation of Textile Dyes by T. hirsuta PW17-41

**Figure 8:**
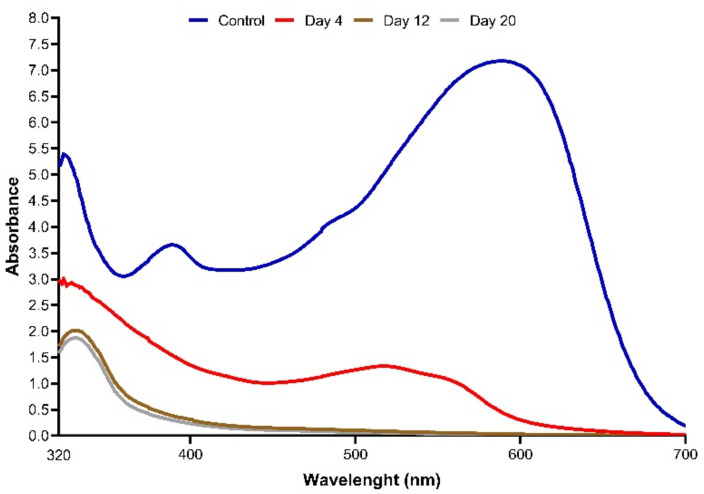
UV–Vis spectra of original textile dyes at day 0 and after treatment by immobilised *T. hirsuta* PW17-41 on days 4, 12 and 20 under optimal conditions.

**Figure 9:**
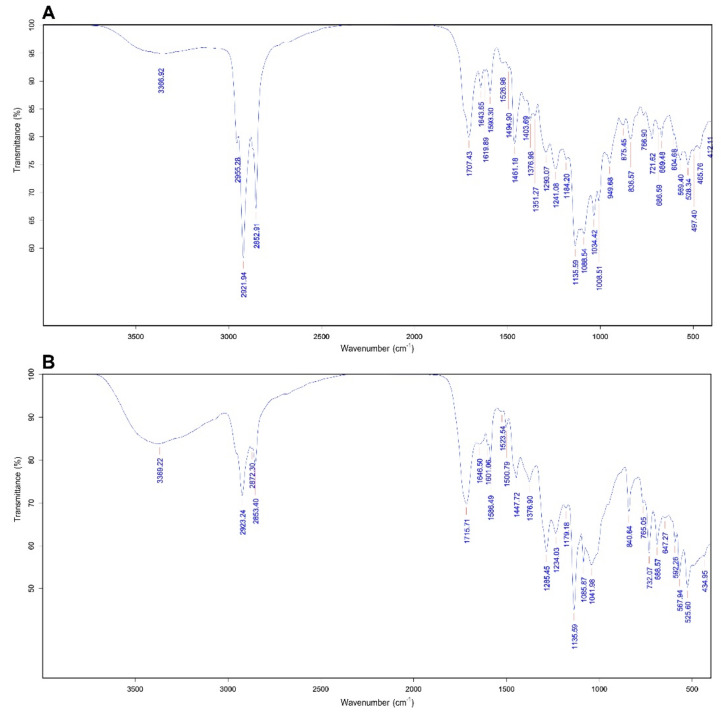
FTIR spectra of original textile dyes (**A**) and decolourised dyes after 12 days. (**B**) Treatment with immobilised *T. hirsuta* PW17-41.

The spectrophotometric scanning in the range of 320–700 nm with original textile dyes and different times of treatments were analysed. The results revealed that the dominant peak appeared at a wavelength of 590 nm on day 0, close to the lambda max values of NEC-R (A608), NR-S3B (A545) and NSB-G (A600) (Figure 8). After treatment with immobilised *T. hirsuta* PW17-41, the intensity of the major peak rapidly decreased on day 4 and completely vanished on days 12 and 20 of the incubation period. This result was confirmed by FTIR analysis of the original textile dyes and after treatment for 12 days. The original spectra presented several peaks, especially at 2955.28 (C-H), 2921.94 (C-H), 2852.91 (C-H), 1707.43 (C=O), 1135.59 (S=O) and 1088.57 (C-N) (Figure 9 and Table S10), which matched the spectra of NEC-R and NSB-G [67]. The transmittance and peak patterns were slightly changed after the decolourisation process. Several peaks decreased or made the stretching vibrations disappear, such as at 1403.69 (C-H), 1351.27 (S+O), 1008.51 (C-O), 949.68 (C-H) and 875.45 (C-H). The difference in the FTIR spectrum confirmed the biodegradation or transmission of textile dye structures [67,68].

## 4. Conclusions

A total of 16 isolates of white-rot fungi belonging to 7 genera (*Amauroderma*, *Coriolopsis*, *Dentipellis*, *Ganoderma*, *Microporus*, *Pseudolagarobasidium*, *Trametes*) and 13 species showed different levels of efficiency of textile dye decolourisation, varying from 28.3 to 74.0%. The highest decolourisation efficiency was obtained in the presence of palm sugar and ammonium nitrate as carbon and nitrogen sources, respectively, with an agitation speed of 100 rpm and a pH of 5. The decolourisation by immobilised *T. hirsuta* PW17-41 was mainly due to the biodegradation process of extracellular MnP and in combination with laccase enzymes, with the decolourisation percentage being higher than 98%. The dye was almost totally decolourised within 2 days when the supplemented dye concentration was 2475 ADMI. Additionally, the immobilised cells could be reused up to 12 times and were still able to remove the textile dyes to lower than 300 ADMI. UV–visible and FTIR spectrum results confirmed the biodegradation of textile dyes during the decolourisation process. Remarkably, the present study used real textile wastewater with high salinity and containing heavy metals, which was evaluated using the ADMI standard values. Interestingly, MnP produced by *T. hirsuta* PW17-41 played an important role in dye decolourisation, which was different from several works that found mainly laccase from genus *Trametes*. Our study adds to the growing body of research suggesting that MnP from immobilised *T. hirsuta* PW17-41 could be a promising eco-friendly alternative to conventional dye removal technologies, with the capacity to produce enzymes that are capable of lignin degradation. A successful phase performed at a bioreactor scale operating in the batch mode or continuous operation will be required to achieve future biotechnological applications. Moreover, a combination treatment between fungi and bacteria is also an area of future research because fungi can degrade the complex structures of synthetic dyes to simple structures, which can be consequently degraded by bacteria. Furthermore, the integration of economic and life cycle assessment analyses will be helpful in determining the long-term sustainability of the immobilised *T. hirsuta* PW17-41 product, which could be an environmentally and sustainable direction for wastewater treatment.

## Figures and Tables

**Table 1:** Characteristics of textile dyes from industrial wastewater.

Characters	Wastewater of Textile Dyes	Industrial Effluent Standard	Characters	Wastewater of Textile Dyes	Industrial Effluent Standard
ADMI	60,100	<300	Nickel (mg L^−1^)	0.005	<1.00
pH	10	5.5–9	Barium (mg L^−1^)	0.048	<1.00
Novacron Ruby S3B (%)	0.52	-	Chromium (mg L^−1^)	<0.005	<0.25
Novacron Navy EC-R (%)	1.20	-	Cadmium (mg L^−1^)	<0.005	<0.03
Novacron Super Black G (%)	0.96	-	Lead (mg L^−1^)	<0.005	<0.20
NaCl (mg L^−1^)	70,000	-	Arsenic (mg L^−1^)	<0.005	<0.25
Na_2_CO_3_ (g L^−1^)	5	-	Mercury (mg L^−1^)	<0.001	<0.005
50% NaOH (mL L^−1^)	0.85	-	Selenium (mg L^−1^)	<0.005	<0.02
Zinc (mg L^−1^)	0.052	<5.00	BOD	29	<20
Copper (mg L^−1^)	0.091	<2.00	COD	199	<120
Manganese (mg L^−1^)	0.017	<500			

## Data Availability

Not applicable.

## Supplementary Materials

The following supporting information was downloaded at: https://www.mdpi.com/article/10.3390/microorganisms10050992/s1: Table S1: Lists of white-rot fungi used to screen the decolourisation of textile dyes in the present study. Table S2: Screening of textile dye decolourisation using white-rot fungi under submerged condition. Table S3: Fungal biomass of immobilised *Trametes hirsuta* PW17-41 on different supports. Table S4: Effects of carbon, nitrogen, pH and agitation speed on the textile dye decolourisation by immobilised *T. hirsuta* PW17-41. Table S5: Effects of the initial textile dye concentrations on the textile dye decolourisation by immobilised *T. hirsuta* PW17-41. Table S6: Adsorption of dead and living biomass of immobilised *T. hirsuta* PW17-41 for textile dye decolourisation. Table S7: Time course study of textile dye decolourisation and MnP and laccase production by immobilised *T. hirsuta* PW17-41 under submerged cultivation conditions using 33% (*v/v*) dye concentration (or 21,200 ADMI) at 30 °C and 100 rpm for 20 day. Table S8: Purification of MnP from *Trametes hirsuta* PW17-41. Table S9: Repeated batch experiment of textile dye decolourisation for 12 cycles by immobilised *T. hirsuta* PW17-41 under optimal conditions. Table S10: FTIR spectrum of original textile dyes and decolourised dyes after 12 days of treatment by immobilised *T. hirsuta* PW17-41. Figure S1: Colours of textile dyes before and after the decolourisation process by immobilised *T. hirsuta* PW17-41 under submerged cultivation conditions. Figure S2: Repeated batch experiment of textile dye decolourisation for 12 cycles by immobilised *T. hirsuta* PW17-41 under optimal conditions.
