# Supplementary material for: High Potential Decolourisation of Textile Dyes from Wastewater by Manganese Peroxidase Production of Newly Immobilised Trametes hirsuta PW17-41 and FTIR Analysis
Source: Microorganisms. 2022 May 9;10(5):992. doi: 10.3390/microorganisms10050992 (PMC9143691; doi:10.3390/microorganisms10050992)
Supplement: Supplementary file 1 [file microorganisms-10-00992-s001.zip › microorganisms-1688331-supplementary.pdf]

Table S1. Lists of white-rot fungi used to screen the decolourisation of textile dyes in the present study.

Taxa	Isolation ID	Location	GenBank Accession number of ITS*
Amauroderma subresinosum	NP17-12	Nakhon Phanom province, Thailand	OM996018
Coriolopsis aspera	NP17-02	Nakhon Phanom province, Thailand	MK589268
C. aspera	NP17-08	Nakhon Phanom province, Thailand	MK589269
C. retropicta	PW17-134	Chaiyaphum province, Thailand	MK589270
Dentipellis parmastoi	PW17-136	Chaiyaphum province, Thailand	MK589290
Ganoderma fornicatum	PW17-145	Chaiyaphum province, Thailand	MK589271
G. lingzhi	PW17-43	Chaiyaphum province, Thailand	MK589272
G. mastoporum	PW17-06	Chaiyaphum province, Thailand	MK589273
G. mastoporum	PW17-154	Chaiyaphum province, Thailand	MK589275
Microporus vernicipes	PW17-173	Chaiyaphum province, Thailand	MK589280
M. xanthopus	PP17-17	Sakhon Nakhon province, Thailand	MK589281
M. xanthopus	PP17-20	Sakhon Nakhon province, Thailand	MK589282
Pseudolagarobasidium sp.	PP17-33	Sakhon Nakhon province, Thailand	MK589289
Trametes elegans	PP17-06	Sakhon Nakhon province, Thailand	MK589285
T. hirsuta	PP17-41	Sakhon Nakhon province, Thailand	MK589286
T. sanguinea	PP17-18	Sakhon Nakhon province, Thailand	MK589287

* ITS = Internal Transcribed Spacers

Table S2. Screening of textile dye decolourisation using white-rot fungi under submerged condition.

**Taxa**	**Isolation ID**	**% Dye decolourisation***
Amauroderma subresinosum	NP17-12	43.52±1.57cde
Coriolopsis aspera	NP17-02	45.12±2.09cd
C. aspera	NP17-08	35.93±6.83ef
C. retropicta	PW17-134	45.51±5.27c
Dentipellis parmastoi	PW17-136	28.26±1.50f
Ganoderma fornicatum	PW17-145	48.24±2.52c
G. lingzhi	PW17-43	50.73±1.25c
G. mastoporum	PW17-06	42.33±0.55cde
G. mastoporum	PW17-154	49.12±1.03c
Microporus vernicipes	PW17-173	28.81±3.09f
M. xanthopus	PP17-17	36.14±0.82def
M. xanthopus	PP17-20	29.31±2.69f
Pseudolagarobasidium sp.	PP17-33	70.88±2.59a
Trametes elegans	PP17-06	44.58±4.56cde
T. hirsuta	PP17-41	73.98±0.74a
T. sanguinea	PP17-18	60.18±2.86b

* values of mean ± standard deviation from triplicate. Letters a-f indicate statistically significant differences between groups according to one-way ANOVA ($n = 3$, $p < 0.05$)

Table S3. Fungal biomass of immobilised *Trametes hirsuta* PW17-41 on different supports.

**Support**	**Fungal biomass of *T. hirsuta* PW17-41 (g)**[1]
nylon sponge	0.375±0.013a
loofah	0.255±0.021b
cascara coffee	0.001±0.000d
parchment coffee	0.099±0.007c

[1]Values are the mean of three replications ± standard deviation. Different letters indicate statistically significant differences between supporters analysed by one-way ANOVA ($n = 3$, $p < 0.05$).

.

Table S4A. Effect of carbon source on decolourisation of textile dyes by immobilisation of *T. hirsuta* PW17-41 at 4% (v/v) concentration after 48 h.

Carbon	% Dye decolourisation*
Fructose	72.34±0.56d
Lactose	76.16±0.36c
Galactose	77.31±1.22bc
Glucose	78.19±0.31ab
Palm sugar	79.47±0.25a
Soluble starch	79.28±0.36ab
Sucrose	78.61±0.16ab
Control	64.98±0.47e

* values of mean ± standard deviation from triplicate. Letters a-e indicate statistically significant differences between groups according to one-way ANOVA ($n = 3$, $p < 0.05$)

Table S4B. Effect of nitrogen source on decolourisation of textile dyes by immobilisation of *T. hirsuta* PW17-41 at 4% (v/v) concentration after 48 h.

Nitrogen	% Dye decolourisation*
Peptone	77.37±0.98bc
Yeast extract	72.54±0.18c
Sodium nitrate	61.33±0.31d
Ammonium nitrate	87.81±4.58a
Ammonium sulphate	82.05±4.03b
Urea	60.30±0.21d
Control	63.80±0.58d

* values of mean ± standard deviation from triplicate. Letters a-d indicate statistically significant differences between groups according to one-way ANOVA ($n = 3$, $p < 0.05$)

Table S4C. Effect of pH on decolourisation of textile dyes by immobilisation of *T. hirsuta* PW17-41 at 4% (v/v) concentration after 48 h.

pH	% Dye decolourisation*
4	84.57±0.21a
5	84.40±0.43a
6	83.42±0.79b
7	59.79±0.60c
8	11.72±0.29d
9	7.60±0.12e
10	6.58±0.41f

* values of mean ± standard deviation from triplicate. Letters a-f indicate statistically significant differences between groups according to one-way ANOVA ($n = 3$, $p < 0.05$)

Table S4D. Effect of agitation speed on decolourisation of textile dyes by immobilisation of *T. hirsuta* PW17-41 at 4% (v/v) concentration after 48 h.

Agitation speed (rpm)	% Dye decolourisation*
0	45.01±2.79d
50	68.80±1.98c
100	94.60±0.31a
150	83.18±1.43b

* values of mean ± standard deviation from triplicate. Letters a-c indicate statistically significant differences between groups according to one-way ANOVA ($n = 3$, $p < 0.05$)

Table S5. Effects of the initial textile dye concentrations on the textile dye decolourisation by immobilised *T. hirsuta* PW17-41.

Dye concentration % (v/v)	Initial ADMI values	% Dye decolourisation*
4	2,450	96.54±0.10a
8	6,100	96.53±0.05a
17	11,150	96.51±0.05a
25	15,350	96.46±0.07ab
33	21,200	96.43±0.12ab
42	26,900	96.20±0.06ab
50	31,700	96.07±0.09b
66	40,800	94.38±0.26c
83	47,700	92.95±0.30d
100	60,100	89.17±0.09e

* values of mean ± standard deviation from triplicate. Letters a-e indicate statistically significant differences between groups according to one-way ANOVA (n = 3, $p < 0.05$)

Table S6. Adsorption of dead and living biomass of immobilised *T. hirsuta* PW17-41 for textile dye decolourisation.

Materials	% Dye decolourisation
Nylon sponge	3.06±0.54e
Mycelium	20.48±0.99d
Dead biomass of immobilised mycelia (adsorption)	28.26±0.37c
Living biomass of immobilised mycelia (degradation)	60.23±0.25b
Living biomass of immobilised mycelia (adsorption+degradation)	89.10±0.23a

* values of mean ± standard deviation from triplicate. Letters a-e indicate statistically significant differences between groups according to one-way ANOVA (n = 3, $p < 0.05$)

Table S7. Time course study of textile dye decolourisation and MnP and laccase production by immobilised *T. hirsuta* PW17-41 under submerged cultivation condition using 33% (v/v) dye concentration (or 21,200 ADMI) at 30°C, 100 rpm for 20 day.

Day	ADMI value	% Dye decolourisation*	MnP (U/L)*	Laccase (U/L)*	Biomass (mg)*	pH*
0	21,200	0.00±0.00f	0.00±0.00f	0.00±0.00f	199.63±7.42a	5.00±0.00a
2	7,653	63.90±1.98e	141.04±16.79f	63.75±6.56f	201.43±0.59a	3.37±0.03b
4	3,142	85.18±0.51d	288.18±42.26f	67.38±2.23f	210.07±6.85a	3.19±0.04c
6	2,032	90.42±0.16c	1,026.89±21.30e	481.48±7.61e	211.80±15.55a	3.19±0.01c
8	866	95.91±0.22b	2,328.89±306.23d	876.67±101.30d	203.00±16.40a	3.16±0.01c
10	493	97.67±0.10ab	2,759.56±191.84cd	1,433.33±164.08c	204.20±15.36a	3.09±0.02c
12	444	97.91±0.03a	3,481.78±283.23b	1,806.42±110.85b	204.87±1.97a	2.81±0.1d
14	398	98.12±0.20a	3,645.33±323.93b	1,902.72±24.81b	205.10±14.20a	2.73±0.04de
16	313	98.52±0.01a	4,942.22±285.65a	2,389.14±102.59a	206.67±12.96a	2.63±0.1de
18	305	98.56±0.01a	4,472.89±331.82a	2,062.96±139.91b	200.73±10.31a	2.63±0.1e
20	300	98.59±0.01a	3,082.22±74.00bc	1,353.09±131.63c	209.07±10.31a	2.59±0.06e

* values of mean ± standard deviation from triplicate. Letters a-f indicate statistically significant differences between groups according to one-way ANOVA (n = 3, $p < 0.05$)

Table S8. Purification of MnP from *Trametes hirsuta* PW17-41

Purification	Volume (mL)	Protein content (mg mL^{-1})	Enzyme activity (U mL^{-1})	Total protein (mg)	Total enzyme activity (U)	Specific activity (U mg^{-1})	Purification fold	Yield (%)
Crude extract	600	0.009	0.715	5.149	429.000	83.314	1	100
$(NH_4)_2SO_4$ precipitation	30	0.034	13.360	1.023	400.800	391.96	4.705	93.427
Dialysis	22	0.040	16.367	0.891	360.067	404.247	4.852	83.932

Table S9. Repeated batch experiment of textile dye decolourisation for 12 cycles by immobilised *T. hirsuta* PW17-41 under optimal conditions.

Cycle	% Dye colourisation*	MnP (U/L)*	Laccase (U/L)*	pH*
1	89.39±0.20c	508.00±122.00e	175.85±10.80f	3.35±0.02bcd
2	89.48±0.29c	1,634.67±268.57de	532.59±80.86def	3.27±0.09cd
3	93.91±0.96ab	4,888.89±763.30bc	1,608.40±207.05abc	3.27±0.12cd
4	94.76±0.99ab	6,548.89±1,293.30ab	1,925.31±85.87ab	3.09±0.03d
5	95.81±0.92a	6,822.22±697.74ab	1,908.03±242.23ab	2.57±0.09e
6	95.70±0.23a	7,151.11±1,276.80a	2,220.99±387.65a	2.68±0.11e
7	95.77±0.44a	5,591.11±692.76abc	1498.77±384.26bcd	3.27±0.21cd
8	95.21±0.05ab	4,842.44±434.18bc	1,218.83±142.11cde	3.31±0.04bcd
9	94.79±0.57ab	4,796.67±547.02bc	1,198.46±14.44cde	3.54±0.20abc
10	93.20±1.83ab	4,000.00±213.78c	935.80±49.39cde	3.55±0.07abc
11	92.74±1.69b	3,840.00±231.79cd	875.62±21.95de	3.70±0.09a
12	92.61±1.21b	3,850±306.68cd	844.14±36.60e	3.63±0.15ab

* values of mean ± standard deviation from triplicate. Letters a-f indicate statistically significant differences between groups according to one-way ANOVA ($n = 3$, $p < 0.05$)

Table S10. FTIR spectrum of original textile dyes and decolourised dyes after 12 days of treatment by immobilised *T. hirsuta* PW17-41.

Spectra		% Transmittance	
Day 0	Day 12	Day 0	Day 12
3,366.92	3,369.22	94.89	83.83
2,955.28	ND	78.85	ND
2,921.94	2,923.24	58.36	71.80
ND	2,872.30	ND	82.67
2,852.91	2,853.40	67.09	79.05
1,707.43	1,715.71	79.93	69.72
1,643.65	1,646.50	88.85	83.73
1,619.89	1,601.06	91.75	83.43
1,593.30	1,586.49	87.81	80.00
1,526.96	1,523.54	93.22	91.33
1,494.90	1,500.79	92.29	87.70
1,461.18	1,447.72	78.81	75.83
1,403.69	ND	86.31	ND
1,376.98	1,376.90	82.92	75.02
1,351.27	ND	83.76	ND
1,293.07	1,285.45	77.24	58.62
1,241.08	1,234.03	74.28	62.89
1,184.20	1,179.18	75.97	68.84
1,135.59	1,135.59	60.44	45.00
1,088.54	1,085.87	62.52	56.02
1,034.42	1,041.98	65.81	55.55
1,008.51	ND	68.50	ND
949.68	ND	75.29	ND
875.45	ND	82.16	ND
836.57	840.64	79.67	68.00
766.90	765.05	83.91	70.07
721.62	732.07	79.64	58.43
686.59	688.57	81.40	60.38
669.48	647.27	79.93	66.50
604.68	592.26	80.36	60.80
569.40	567.94	75.83	53.04
528.34	525.60	75.04	50.33
497.40	ND	77.46	ND
465.76	434.95	77.89	57.57
412.11	ND	82.66	ND

ND = not detected

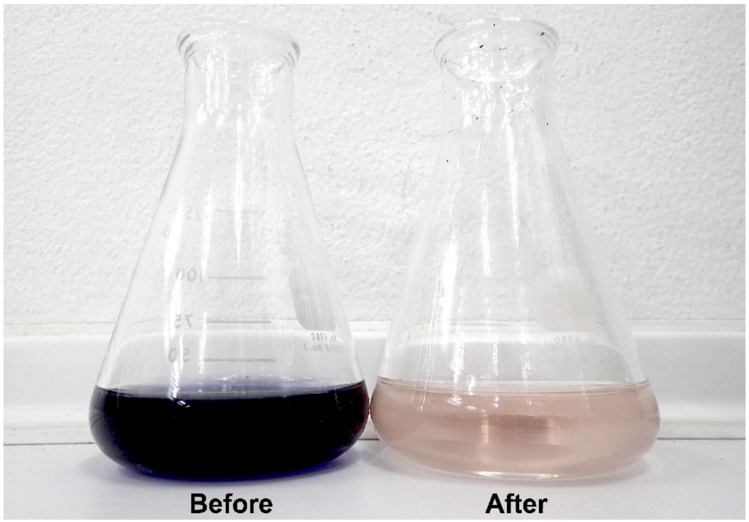

Figure S1. Colours of textile dyes before and after the decolourisation process by immobilised *T. hirsuta* PW17-41 under submerged cultivation condition.

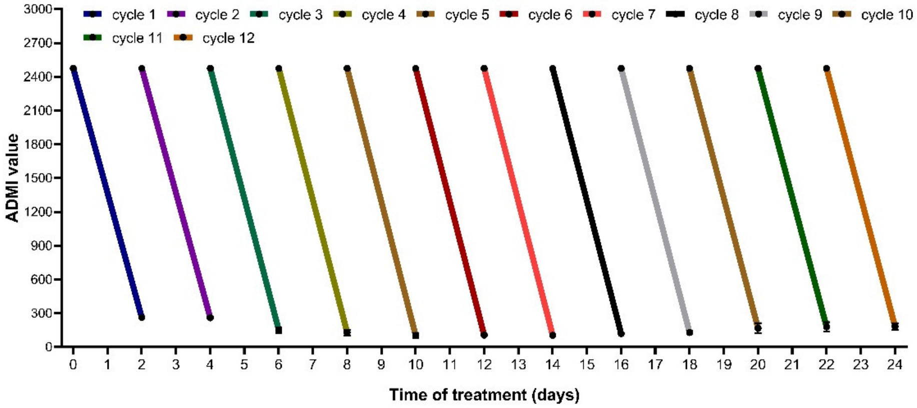

Figure S2: Repeated batch experiment of textile dye decolourisation for 12 cycles by immobilised *T. hirsuta* PW17-41 under optimal conditions.
